# Treatment received and treatment adequacy of depressive disorders among young adults in Finland

**DOI:** 10.1186/s12888-015-0427-8

**Published:** 2015-03-11

**Authors:** Teija Kasteenpohja, Mauri Marttunen, Terhi Aalto-Setälä, Jonna Perälä, Samuli I Saarni, Jaana Suvisaari

**Affiliations:** 1 Department Health, Mental Health Unit, National Institute for Health and Welfare, P.O. Box 30,, 00271 Helsinki, Finland; 2 Adolescent Psychiatry, University of Helsinki and Helsinki University Hospital, Helsinki, Finland; 3 The Social Insurance Institute, Helsinki, Finland; 4 Turku University Hospital and University of Turku, Turku, Finland; 5 Department of Social Psychiatry, Tampere School of Public Health, University of Tampere, Tampere, Finland

**Keywords:** Depressive disorders, Treatment, Young adults

## Abstract

**Background:**

Under-treated depression may be especially harmful in early adulthood. The aims of this study are to describe treatments received for depressive disorders, to define factors associated with treatment adequacy and dropouts from treatment in a Finnish general population sample of young adults.

**Methods:**

A nationally representative two-stage cluster sample of 1894 Finns aged 19 to 34 years was sent a questionnaire containing several mental health screens. All screen positives and a random sample of screen negatives were invited to participate in a mental health assessment including a SCID interview. Case records from mental health treatments for the same sample were obtained for the final diagnostic assessment. Based on all available information, receiving antidepressant pharmacotherapy for at least two months with at least four visits with any type of physician or at least eight sessions of psychotherapy within 12 months or at least four days of hospitalization were regarded as minimally adequate treatment. Treatment dropout was rated if the treatment strategy was assessed to be adequate according to the case records but the patient discontinued the visits.

**Results:**

Of participants with depressive disorders (n = 142), 40.9% received minimally adequate treatment. In multiple logistic regression models, substance use disorder and female gender were associated with at least one visit with a physician, while having major depressive disorder was associated with visits with a physician at least 4 times a year. Women had higher odds of having received any psychotherapy and psychotherapy lasting for at least 8 sessions in a year. Low education and a history of suicide attempt were associated with increased odds of treatment dropout. None of the factors explained the final outcome of minimally adequate treatment.

**Conclusions:**

Treatment adequacy in the present study was better than previously seen, but more efforts are needed to provide adequate treatment for young adults, especially those with low education and suicidality.

**Electronic supplementary material:**

The online version of this article (doi:10.1186/s12888-015-0427-8) contains supplementary material, which is available to authorized users.

## Background

Depressive disorders are a leading cause of burden of disease worldwide [1]. Mood disorders are also the most costly disorders and depression the most burdensome disorder of the brain in Europe, mainly because of high indirect costs caused by disability [2-4]. However, depressive disorders are still often unrecognized and under-treated despite existing forms of effective treatment [5-7].

Untreated mental disorders are particularly harmful in adolescence and early adulthood, since they limit work, educational ability, and social interaction in a critical life-stage of identity formation and socialization [8]. Though the prevalence of depression is at its peak during this particular period [9-11], studies that focus on young adults and depressive disorders and their treatment are relatively rare. In the National Comorbidity Survey Replication, Kessler also investigated the group aged 18–29 and found a lifetime prevalence of 15.4% for major depressive disorder and 1.7% for dysthymia [10]. Aalto-Setälä et al. presented a prevalence of 14.9% for twelve-month depressive disorders among young urban adults in Finland aged 20–24 in 1995. About half of them had had a contact with mental health services and about one-third had reported treatment contacts during the index episode [12]. Haarasilta et al. found that about half of the subjects with MDE were estimated to be in need of treatment but only 20.6% had sought care for depression during the preceding year in a sample of 15–24 year olds from the Finnish Health Care Survey of 1996 [13].

Studying adequate treatment is important because guideline concordant depression care is associated with better patient outcome [14,15]. However, Hepner et al. found that primary care physicians could often detect depression and initiate treatment but had difficulties in completion of a minimal course of treatment for depression [15]. Eisenberg et al. in turn investigated college students in the United States and found only 22% of depressed students received minimally adequate treatment [16].

Previous studies have shown that mental health treatment dropout is a common problem [17-20] that may limit treatment effectiveness [21,22] and be a sign of poor clinical performance [23]. Therefore, understanding how different risk factors are associated with dropout is important for designing mental health services.

This study is based on a nationwide, representative population-based sample of young Finnish adults aged 20–34. The aims of this study are to describe treatment received for depressive disorders, treatment adequacy and dropouts from treatment among young adults; to investigate sociodemographic correlates; and to identify comorbid mental disorders and disorder-specific factors that possibly affect treatment adequacy and dropout.

## Methods

### Sample

Data were derived from the Mental Health in Early Adulthood in Finland (MEAF) study. Methods have been reported in detail elsewhere [24]. MEAF is based on a nationally representative two-stage cluster sample (n = 1894) of young adults aged 18–29 years in the Health 2000 study, which was a comprehensive health survey where the original young adult assessment was carried out in 2001. Although there were questions related also to mental health in the Health 2000 young adult protocol, a structured diagnostic interview was not conducted [25-27]. Therefore, a substudy focusing on mental health (MEAF) was launched. Study designs of Health 2000 and MEAF are described in more detail in Additional file 1: Methods.

**Figure 1:**
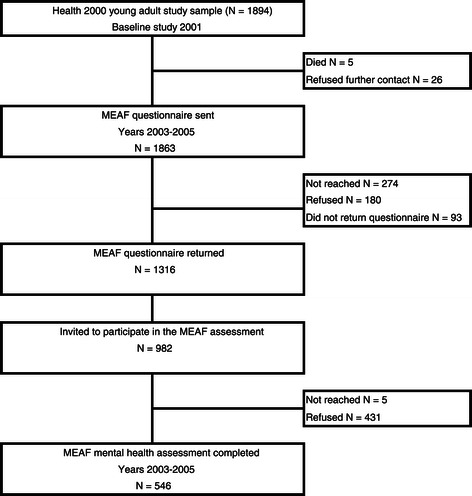
**Mental Health in Early Adulthood in Finland (MEAF) study flow.** Adapted from Suvisaari et al. 2009 [24], courtesy of Cambridge University Press.

The study flow of the MEAF study is presented in Figure 1. A questionnaire was mailed 2–4 years after the original study to all members of the young adult sample, excluding those who had died or refused further contacts. Participants were aged 20–34 during this time period. The questionnaire included several scales assessing mental health and substance use: K10 [28] and the GHQ-12 [29] for general psychological distress, SCOFF [30] for eating disorders, 22 questions on delusions and hallucinations for psychotic disorders from the CIDI interview [31], the MDQ [32] for manic symptoms, and CAGE [33] for alcohol abuse. Persons who reported symptoms above a defined threshold in any screening scale were asked to participate in the mental health interview. Information from the Finnish National Hospital Discharge Register (NHDR) was used to identify all persons who had received hospital treatment due to any mental disorder [International Classification of Diseases (ICD)-10 section F, ICD-8 and ICD-9 290–319] and they were asked to participate in the interview. In addition, a random subsample of Health 2000 young adults was invited to the interview regardless of their answers to the screening questionnaire. Persons selected via NHDR who had not returned the MEAF questionnaire were contacted through the person responsible for the treatment, usually their general practitioner or psychiatrist. Screening instruments and their cut-off points are shown in Table 1. The screening procedure and selection of the cut-off points for each screen are described in detail in Additional file 1: Methods. [24].

**Table 1:** **Screens used for selecting persons to mental health interview**

Screen	Symptoms that the screen assesses	Cut-off point or criterion for selection	Number (%) of persons selected by the screen
**General Health Questionnaire (GHQ-12)**	Psychological distress (past month)	>3	245 (18.6%)^a^
**K10**	Psychological distress (past month)	>18	215 (16.3%)^a^
**SCOFF**	Eating disorders (current)	>1	127 (9.7%)^a^
**CIDI section G (psychotic symptoms)**	Psychotic disorders (lifetime)	At least one symptom	348 (26.4%)^a^
**Mood Disorder Questionnaire (MDQ)**	Bipolar spectrum disorders (lifetime)	>6	170 (12.9%)^a^
**CAGE**	Alcohol use disorders (lifetime)	>2	229 (17.4%)^a^
**Use of any illicit drug**	Substance use disorders (lifetime)	At least 6 times	98 (7.4%)^a^
**Suicide attempt**	Severe suicidality	At least one attempt	46 (3.5%)^a^
**Use of health services for mental health problem**	All lifetime disorders	At least once	239 (18.2%)^a^
**Perceived need for treatment**	All lifetime disorders	Self-reported need for treatment	90 (6.8%)^a^
**Hospitalization due to any mental health disorder**	All lifetime disorders	ICD-10 group F or ICD-8 and ICD-9 290-319	120 (6.3%)^b^

^a^N = 1316 (participants who returned a questionnaire including screens).

^b^N = 1894 (all participants aged 18–29 years in the Health 2000 study).

The ethics committees of the National Public Health Institute (since 2009 the National Institute of Health and Welfare) and the Hospital District of Helsinki and Uusimaa approved the Health 2000 survey and the MEAF reassessment. Participants provided written informed consent [24,25].

### Mental health assessment

The mental health interview was conducted using the research version of the Structured Clinical Interview for DSM-IV (SCID-I) [34]. The sections on mood, psychotic, substance use, anxiety and eating disorders were included in the assessment. The SCID Screening module was used at the beginning of the interview to enhance reliability [35].

The assessment began with a neuropsychological test battery [36] followed by the mental health interview. The interview started with questions on sociodemographic factors and treatment received for mental health problems, followed by the SCID-I interview and questions assessing the lifetime occurrence of suicidal ideation and behavior, the Global Assessment of Functioning (GAF), and the Social and Occupational Functioning Assessment Scale (SOFAS) [37]. The assessments were carried out by experienced research nurses or psychologists who attended a one-week training period and regular follow-up sessions. All interviews were reviewed by the interviewer together with a psychiatrist.

Another questionnaire was given to participants after the interview. The post-interview questionnaire sought further information on the person’s mental health and associated factors.

### Final diagnostic assessment

For the final diagnostic assessment, all case records from hospital and outpatient treatment were obtained with the participant’s approval. Permission to view the case records of non-participants was obtained from the Finnish Ministry of Social Affairs and Health, excluding those who had refused any participation in the Health 2000 study. Case records were compiled using information from the Hospital Discharge Register, self-reported mental health care contacts, and primary health care centers. The aim was to gather information on all lifetime treatments for mental health disorders.

The final best-estimate diagnoses using DSM-IV-TR criteria were made by four experienced clinicians (J.S., T.A.-S., S.S., J.P.). Diagnostic assessment was based on all available systematically evaluated information from the interview and/or case records. Disorders not covered by SCID-I were also evaluated, including personality disorders. The reliability of the diagnoses was tested on 40 cases rated by all four clinicians. Unweighted Kappa values between each pair of raters ranged from 0.94 to 1.00 for major depressive disorder and from 0.90 to 1.0 for any depressive disorder [24].

The lifetime prevalence of depressive disorders (i.e. major depressive disorder, dysthymia or depressive disorder NOS) in this sample was 17.7% [24]. This paper investigates this subgroup which consisted of 142 participants after excluding those with a diagnosis of schizophrenia spectrum disorders (3 persons).

### Use of mental health services and treatment received

From mental health interviews and case records we gathered information on mental health care use as well as treatment received. All data were collected on both the most recent and the most intensively treated episode of depression. It turned out, however, that they differed only in 18 cases (12.7%). Our aim was to investigate the average as well as the most recent functioning of the health care system. Therefore, the present paper focuses on treatment received during the most recent depressive episode. The distribution of sociodemographic and disorder-specific factors, comorbid psychiatric disorders and treatments received and dropouts during the most intensively treated depressive episode are available in the Additional file 2: Table S1, Additional file 3: Table S2 and Additional file 4: Table S3.

We determined criteria for minimally adequate treatment according to evidence-based guidelines [38,39]. Based on this information, receiving antidepressant pharmacotherapy for at least two months with at least four visits with any type of physician or at least eight sessions of psychotherapy within 12 months were regarded as minimally adequate treatment. The same criteria were used in the European Study of the Epidemiology of Mental Disorders and the National Comorbidity Survey Replication [40,41]. We also defined at least four days of hospitalizations for depression as adequate care. The criteria are described in detail in Additional file 1: Methods.

Visits with physicians related to depressive symptoms were assessed from case records and interviews and divided into three categories: none, at least one visit or at least four visits within 12 months. We also included in visits a telephone consultation between a patient and physician as well as consultations between health care professionals and the treating physician that concerned the patient’s treatment.

A psychotherapeutic session was defined as a visit with a psychiatrist, psychologist or psychotherapist in all settings or a visit with any professional in a psychiatric clinic. We counted the sessions of psychotherapy and divided them into three categories: none, at least one session, or at least eight sessions within a year.

Pharmacotherapy was evaluated for the type and duration. The information about antidepressant use was divided into three categories: not prescribed, prescribed or used for at least two months. We also gathered information about hospitalizations, their cause and duration.

We rated treatment dropout if the treatment strategy was assessed to be adequate according to the case records but the patient discontinued the visits by his own decision. A definition of treatment dropout is described in more detail in Additional file 1: Methods.

Case records were ordered from 99 participants with depressive disorder based on either self-reported outpatient treatment contact or register information on hospital treatments. They were received for 86 (86.9%) of them, whereas in 13 cases the records were not found. In these cases, the quality of care was coded based on information from the interview which included questions about the type, frequency and duration of treatment.

### Sociodemographic, disorder-specific and comorbid factors

Information on sociodemographic and disorder specific factors and comorbid psychiatric disorders was gathered in the mental health interview and from case records. Based on this information, we also defined broadly the start and end points for depressive episodes and formed new variables concerning the duration of depression and age at the onset of illness.

We studied the relationship between sociodemographic factors and treatment using the following variables: gender, marital status, age at the time the MEAF-questionnaire was sent (19–24 years, 25–29 years and 30–34 years old), basic education (less than high school/high school) and current employment (employed, student, unemployed, other). High school means twelve years of education followed by a matriculation examination. For the “other” employment group, 5.6% (1) were on disability pension and 94.4% (17) were at home taking care of household and family members. We examined the effect of basic education only, since some of the younger members of the cohort had not yet finished their vocational or higher education.

We studied whether disorder-specific factors such as the severity of disease (i.e. MDD or not) and duration of depression affected the treatment adequacy. The effect of a lifetime history of suicide attempts was examined based on self-reported suicide attempts in the questionnaire and/or interview or suicide attempts according to case records, as described by Suokas and collegues [42]. The impact of comorbid psychiatric disorders on treatment was examined for the other three main categories of non-psychotic disorders: anxiety disorders, substance use disorders, and eating disorders.

### Statistical analysis

We used standard statistical methods to generate descriptive statistics. Since the analysis was limited to the subgroup of depressive participants, we did not use survey weights in the analysis. The associations of the following sociodemographic factors with treatments received were analyzed: gender, age, basic education, employment and marital status. We also examined whether suicidality, duration of depressive episode, having a diagnosis of MDD, or comorbid psychiatric disorders affected the treatment adequacy. The relationship of all these variables was examined separately for different components of care, i.e. pharmacotherapy, physician visits, psychotherapy and finally the overall treatment adequacy. We also examined what kind of patients had the highest risk of discontinuing treatment. Differences were tested using the *χ*^2^ or Fisher’s exact tests when appropriate. Logistic regression analyses were used to identify variables that were independently associated with the use of mental health services and the type and adequacy of treatment. Gender, age, age at the onset of depression (continuous), basic education, history of suicidal attempts, diagnosis of MDD, comorbid anxiety disorder and substance use disorder were entered simultaneously into a logistic regression model to explore the factors affecting treatment. Values of p < 0.05 were considered statistically significant. The SAS 9.3 statistical package was used in the analyses.

## Results

### Participants

Table 2 shows the socio-demographic, disorder-specific and comorbid psychiatric disorder factors of the participants. Male participants with any diagnosis of life-time depressive disorder numbered 45 (31.7%) and female participants 97 (68.3%). There was a statistically significant difference in basic education and current employment between the genders. Of the comorbid psychiatric disorders men had more substance use disorders and women had more eating disorders. A median time between the beginning of the last depressive episode and the survey was 2.3 years, ranging from 0 to 23.6 years. 10.6% (15) of participants were still (during the last month before the interview) in their most recent episode.

**Table 2:** **Socio-demographic and disorder specific factors and comorbid psychiatric disorders of participants**

		All		Men		Women		
		100.0%	N = 142	31.7%	N = 45	68.3%	N = 97	
Variable	Category	%	N	%	N	%	N	p ^ b ^
**Age**	**<25 years**	21.8	31	20.0	9	22.7	22	
	**25-29 years**	37.3	53	40.0	18	36.1	35	
	**≥30 years**	40.9	58	40.0	18	41.2	40	0.8873
**Basic education**	**Less than high school**	50.7	69	64.1	25	45.4	44	
	**High school**	49.3	67	35.9	14	54.6	53	**0.0480**
**Married or cohabiting**	**Yes**	50.4	69	42.5	17	53.6	52	
	**No**	49.6	68	57.5	23	46.4	45	0.2371
**Current employment**	**Employed**	58.4	80	70.0	28	53.6	52	
	**Student**	21.2	29	15.0	6	23.7	23	
	**Unemployed**	7.3	10	15.0	6	4.1	4	
	**Other** ^**a**^	13.1	18	0.0	0	18.6	18	**0.0025**
**Major depressive disorder** ^**d**^	**Yes**	78.2	111	80.0	36	77.3	75	
	**No** ^**e**^	21.8	31	20.0	9	22.7	22	0.7190
**Suicide attempts**	**Yes**	12.0	17	15.6	7	10.3	10	
	**No**	88.0	125	84.4	38	89.7	87	0.3703
**Duration of depression > 1 year**	**Yes**	33.9	41	29.4	10	35.6	31	
	**No**	66.1	80	70.6	24	64.4	56	0.5158
**Comorbid anxiety disorder** ^**d**^	**Yes**	32.4	46	31.1	14	33.0	32	
	**No**	67.6	96	68.9	31	67.0	65	0.8239
**Comorbid substance use disorder** ^**d**^	**Yes**	19.0	27	42.2	19	8.3	8	
	**No**	81.0	115	57.8	26	91.8	89	**<0.0001**
**Comorbid eating disorder** ^**d**^	**Yes**	8.5	12	0.0	0	12.4	12	
	**No**	91.6	130	100.0	45	87.6	85	**0.0099** ^**c**^

^a^Of the other group, 5.6% (1) were on disability pension and 94,4% (17) at home taking care of household and family members.

^b^The p-values indicate a significance of the difference between genders in the distribution of each catergory tested by *χ*2- or Fisher’s exact test. P-values < 0.05 in boldface.

^c^Fisher’s exact test was used in the analysis.

^d^Diagnosis is a lifetime diagnosis.

^e^Dysthymia or depressive disorder NOS.

### Treatment received and treatment adequacy

Of all participants with depressive disorder, 76.1% had had some kind of contact with the health care system for their depression. More than two thirds had visited a physician and 28.5% had had at least four visits within a year. Antidepressive medication was prescribed to 43.7% and almost a third had used antidepressants for at least two months. Guideline-concordant pharmacotherapy was received by 17.6% of participants. Almost 60% of subjects had attended psychotherapy sessions, a third at least eight times a year. Any hospital treatment was received by 9.2% and at least 4 days of hospitalization by 7.0% of all participants. A total of 40.9% of subjects had received minimally adequate treatment (Table 3).

**Table 3:** **Sociodemographic factors, treatments received and dropouts during the most recent depressive episode** ^**i**^

				Pharmacotherapy				Visits with a physician/a year				Guideline-concordant pharmacotherapy ^ c ^		Sessions of psychotherapy/a year				Minimally adequate treatment ^ e ^		Treatment dropout ^ f ^	
				Any ^ a ^		≥2 months		Any ^ b ^		≥4 times				Any ^ d ^		≥8 times					
Variable	Category		N	%	N	%	N	%	N	%	N	%	N	%	N	%	N	%	N	%	N
**All**			142	43.7	62	31.6	43	67.2	92	28.5	39	17.6	25	59.6	84	33.3	47	40.9	58	15.7	18
**Gender**	**Male**		45	46.7	21	38.1	16	59.1	26	29.6	13	20.0	9	53.3	24	20.0	9	35.6	16	22.9	8
	**Female**		97	42.3	41	28.7	27	71.0	66	28.0	26	16.5	16	62.5	60	39.6	38	43.3	42	12.5	10
		**p** ^**g**^		0.6229		0.2775		0.1670		0.8474		0.6099		0.3012		**0.0215**		0.3824		0.1596	
**Agegroup**	**<25 years**		31	64.5	20	46.7	14	80.7	25	38.7	12	35.5	11	71.0	22	54.8	17	61.3	19	15.4	4
	**25-29 years**		53	28.3	15	19.2	10	52.9	27	27.5	14	13.2	7	54.7	29	26.4	14	32.1	17	14.0	6
	**≥30 years**		58	46.6	27	35.2	19	72.7	40	23.6	13	12.1	7	57.9	33	28.1	16	37.9	22	17.4	8
		**p** ^**g**^		**0.0046**		**0.0280**		**0.0183**		0.3241		**0.0125**		0.3235		**0.0157**		**0.0266**		0.9045	
**Basic**	**Less than high school**		69	47.8	33	32.8	21	76.1	51	32.8	22	17.4	12	60.3	41	33.8	23	43.5	30	22.2	12
**education**	**High school**		67	35.8	24	27.3	18	56.9	37	20.0	13	14.9	10	56.7	38	32.8	22	35.8	24	3.6	2
		**p** ^**g**^		0.1560		0.4908		**0.0193**		0.0949		0.6962		0.6731		0.9031		0.3615		**0.0033**	
**Current**	**Employed**		80	37.5	30	25.0	19	65.4	51	25.6	20	11.3	9	57.0	45	26.6	21	36.3	29	12.5	8
**employment**	**Student**		29	55.2	16	41.4	12	71.4	20	35.7	10	27.6	8	65.5	19	48.3	14	51.7	15	12.0	3
	**Unemployed**		10	20.0	2	11.1	1	40.0	4	10.0	1	10.0	1	30.0	3	10.0	1	10.0	1	37.5	3
	**Other**		18	50.0	9	41.2	7	76.5	13	23.5	4	22.2	4	66.7	12	50.0	9	50.0	9	0.0	0
		**p** ^**g**^		0.1580		0.1542		0.2374		0.4702^h^		0.1566^h^		0.2138		**0.0260**		0.0842		0.1131^h^	
**Married or**	**No**		68	39.7	27	26.2	17	60.0	39	23.1	15	17.7	12	53.7	36	34.3	23	39.7	27	8.8	5
**cohabiting**	**Yes**		69	43.5	30	33.3	22	72.1	49	29.4	20	14.5	10	62.3	43	31.9	22	39.1	27	16.7	9
		**p** ^**g**^		0.6542		0.3689		0.1418		0.4069		0.6151		0.3102		0.7620		0.9451		0.2105	

^a^Antidepressant prescribed.

^b^At least 1 visit with a physician a year.

^c^Antidepressant used for at least 2 months + 4 visits with a physician a year.

^d^At least 1 session of psychotherapy a year.

^e^Antidepressant used for at least 2 months + at least 4 visits with a physician a year or at least 8 sessions of psychotherapy a year or a hospitalization for depressive symptoms lasting for at least 4 days.

^f^A participant discontinued the visits despite adequate treatment plan.

^g^The p-values indicate a significance of the difference between categories in a distribution of treatments and dropout tested by *χ*2- or Fisher’s exact test. P-values < 0.05 in boldface.

^h^Fisher’s exact test was used in the analysis.

^i^A bivariate analysis.

### Socio-demographic factors and treatment received

An association was found between adequate psychotherapy and gender as well as current employment in the bivariate analysis. Women had more often than men attended at least eight sessions of psychotherapy a year. Individuals who were currently students or in the category “other” had more often received psychotherapy at least eight times a year than the rest of the sample (Table 3).

Basic education was statistically significantly associated with appointments with physicians in the bivariate analysis: the proportion of individuals who had no visits with a doctor was higher among those who had completed high school than among those with less education.

Age at the time of the study had an association with different aspects of treatment in the bivariate analysis: the youngest age group had received most forms of treatment as well as minimally adequate treatment more often than the older participants (Table 3).

### Disorder-specific factors, comorbid psychiatric disorders, and treatment received

The relationship between treatment received and disorder-specific factors and comorbidity is presented in Table 4. Having a diagnosis of MDD was related to antidepressant medication for at least two months and physician visits at least four times a year in the bivariate analysis. Duration of depressive episode was related to minimally adequate treatment and appropriateness of care in every aspect.

**Table 4:** **Disorder-specific factors, comorbid psychiatric disorders, treatments received and dropouts during the most recent depressive episode** ^**i**^

				Pharmacotherapy				Visits with physician/a year				Guideline-concordant pharmacotherapy ^ c ^		Sessions of psychotherapy/a year				Minimally adequate treatment ^ e ^		Treatment dropout ^ f ^	
				Any ^ a ^		≥2 months		Any ^ b ^		≥4 times				Any ^ d ^		≥8 times					
Variable	Category		N	%	N	%	N	%	N	%	N	%	N	%	N	%	N	%	N	%	N
**Major**	**Yes**		111	46.9	52	36.1	39	69.8	74	33.0	35	20.7	23	61.8	68	35.5	39	43.2	48	14.8	13
**depressive disorder**	**No**		31	32.3	10	14.3	4	58.1	18	12.9	4	6.5	2	51.6	16	25.8	8	32.3	10	18.5	5
		**p** ^**g**^		0.1476		**0.0269**		0.2206		**0.0290**		0.0651		0.3065		0.3142		0.2713		0.7623^h^	
**Duration of**	**Yes**		41	70.7	29	60.0	24	84.6	33	46.2	18	36.6	15	77.5	31	65.0	26	68.3	28	20.6	7
**episode >1 year**	**No**		80	28.8	23	18.0	14	60.3	47	18.0	14	8.8	7	48.8	39	21.3	17	26.3	21	9.2	6
		**p** ^**g**^		**<.0001**		**<.0001**		**0.0076**		**0.0013**		**0.0002**		**0.0026**		**<.0001**		**<.0001**		0.1276^h^	
**Suicide attempts**	**Yes**		17	58.8	10	41.2	7	81.3	13	43.8	7	23.5	4	76.5	13	17.7	3	29.4	5	60.0	6
	**No**		125	41.6	52	30.3	36	65.3	79	26.5	32	16.8	21	57.3	71	35.5	44	42.4	53	11.4	12
		**p** ^**g**^		0.1791		0.3649		0.2014		0.2360^h^		0.5019^h^		0.1301		0.1435		0.3067		**9.286E-04** ^**h**^	
**Comorbid**	**Yes**		46	50.0	23	42.2	19	77.8	35	37.8	17	23.9	11	67.4	31	34.8	16	43.5	20	21.1	8
**anxiety disorder**	**No**		96	40.6	39	26.4	24	62.0	57	23.9	22	14.6	14	55.8	53	32.6	31	39.6	38	13.0	10
		**p** ^**g**^		0.2918		0.0614		0.0641		0.0912		0.1719		0.1881		0.7995		0.6586		0.2628	
**Comorbid**	**Yes**		27	59.3	16	45.8	11	84.0	21	40.0	10	18.5	5	69.2	18	23.1	6	40.7	11	38.9	7
**substance use disorder**	**No**		115	40.0	46	28.6	32	63.4	71	25.9	29	17.4	20	57.4	66	35.7	41	40.9	47	11.3	11
		**p** ^**g**^		0.0694		0.0989		**0.0473**		0.1576		1.0000^h^		0.2666		0.2193		0.9902		**0.0080** ^**h**^	
**Comorbid**	**Yes**		12	50.0	6	33.3	4	90.9	10	45.5	5	25.0	3	50.0	6	41.7	5	41.7	5	11.1	1
**eating disorder**	**No**		130	43.1	56	31.5	39	65.1	82	27.0	34	16.9	22	60.5	78	32.6	42	40.8	53	16.0	17
		**p** ^**g**^		0.6436		1.0000^h^		0.1013^h^		0.2931^h^		0.4432^h^		0.5458^h^		0.5342^h^		1.0000^h^		1.0000^h^	

^a^Antidepressant prescribed.

^b^At least 1 visit with a physician a year.

^c^Antidepressant used for at least 2 months + 4 visits with a physician a year.

^d^At least 1 session of psychotherapy a year.

^e^Antidepressant used for at least 2 months + at least 4 visits with a physician a year or at least 8 sessions of psychotherapy a year or a hospitalization for depressive symptoms lasting for at least 4 days.

^f^A participant discontinued the visits despite adequate treatment plan.

^g^The p-values indicate a significance of the difference between categories in a distribution of treatments and dropout tested by *χ*2- or Fisher’s exact test. P-values < 0.05 in boldface.

^h^Fisher’s exact test was used in the analysis.

^i^A bivariate analysis.

In the bivariate analysis of comorbid disorders, only substance use disorder was associated statistically significantly with treatment: participants with substance use disorder had at least one visit with a physician more often than others. There was also a trend in the direction of an association between substance use and any pharmacotherapy (p = 0.069). The same kind of trend was seen in the association between comorbid anxiety disorder and having at least two months of pharmacotherapy (p = 0.061) and having at least one visit with a physician (p = 0.064).

In our study group, 15.7% dropped out of treatment (Table 3). Participants with less education, a history of suicide attempts, or substance use disorder interrupted their treatment more often than others according to the bivariate analysis (Tables 3 and 4).

### Factors associated with treatment and dropout in multivariate analyses

In the multivariate analysis having a diagnosis of MDD was associated with increased odds for visits with a physician at least four times a year (OR 5.44, CI 1.40–20.12, P = 0.014) (Table 5). This difference was not seen in the analysis concerning the most intensively treated depressive episode. Instead the participants who had completed high school had lower odds of visiting a physician at least four times a year (Additional file 4: Table S3). Substance use disorder (OR 8.29, CI 1.69–40.58, P = 0.009) and female gender (OR 4.45, CI 1.58–12.54, P = 0.005) were associated with at least one visit with a physician.

**Table 5:** **Logistic regression models of variables associated with treatments received and dropout during the depressive episode** ^**g, f**^

		Pharmacotherapy				Visits with a physician/a year				Guideline-concordant pharmacotherapy ^ c ^		Sessions of psychotherapy/a year				Minimally adequate treatment ^ e ^		Treatment dropout ^ f ^	
		Any ^ a ^		≥2 months		Any ^ b ^		≥4 times				Any ^ d ^		≥8 times					
Variable	Category	OR	95% CI	OR	95% CI	OR	95% CI	OR	95% CI	OR	95% CI	OR	95% CI	OR	95% CI	OR	95% CI	OR	95% CI
**Gender**	**Male (ref.)**	1.00	-	1.00	-	1.00	-	1.00	-	1.00	-	1.00	-	1.00	-	1.00	-	1.00	-
	**Female**	1.42	0.58-3.49	0.89	0.34-2.30	**4.45	1.58-12.54	2.01	0.67-6.01	0.97	0.30-3.13	*2.53	1.04-6.16	*3.37	1.20-9.46	2.15	0.86-5.34	2.67	0.42-17.06
**Agegroup**	**<25 years (ref.)**	1.00	-	1.00	-	1.00	-	1.00	-	1.00	-	1.00	-	1.00	-	1.00	-	1.00	-
	**25-29 years**	**0.20	0.07-0.57	*0.30	0.10-0.92	**0.18	0.05-0.63	0.89	0.28-2.81	0.32	0.09-1.13	0.56	0.20-1.60	0.39	0.14-1.14	0.36	0.13-1.01	1.80	0.23-14.07
	**≥30 years**	0.40	0.12-1.28	0.67	0.20-2.27	0.36	0.09-1.45	0.67	0.18-2.45	0.28	0.06-1.27	0.61	0.18-1.99	0.44	0.13-1.49	0.63	0.20-2.02	2.43	0.23-25.40
**Age at the onset of**	**Continuous**	1.02	0.93-1.12	1.00	0.90-1.11	1.08	0.97-1.20	0.95	0.86-1.06	1.00	0.88-1.14	0.98	0.90-1.08	0.99	0.89-1.09	0.96	0.87-1.05	0.95	0.79-1.14
**depression**																			
**Basic education**	**Less than high school (ref.)**	1.00		1.00	-	1.00	-	1.00	-	1.00	-	1.00	-	1.00	-	1.00	-	1.00	-
	**High school**	0.78	0.36-1.72	1.02	0.43-2.40	0.47	0.20-1.14	0.57	0.23-1.38	1.10	0.39-3.08	1.01	0.47-2.18	0.80	0.36-1.79	0.66	0.30-1.45	*0.15	0.03-0.86
**Major**	**No (ref.)**	1.00	-	1.00	-	1.00	-	1.00	-	1.00	-	1.00	-	1.00	-	1.00	-	1.00	-
**depressive disorder**	**Yes**	1.90	0.74-4.87	2.66	0.81-8.77	2.00	0.77-5.24	*5.44	1.40-21.12	3.21	0.66-15.48	1.79	0.75-4.25	1.40	0.54-3.63	1.59	0.63-3.97	1.21	0.24-6.12
**Suicide attempts**	**No (ref.)**	1.00	-	1.00	-	1.00	-	1.00	-	1.00	-	1.00	-	1.00	-	1.00	-	1.00	-
	**Yes**	1.14	0.33-3.86	0.84	0.23-3.15	0.88	0.19-4.08	1.89	0.51-6.96	1.65	0.36-7.66	1.74	0.47-6.46	0.43	0.11-1.80	0.39	0.10-1.43	*6.73	1.10-41.17
**Comorbid**	**No (ref.)**	1.00	-	1.00	-	1.00	-	1.00	-	1.00	-	1.00	-	1.00	-	1.00	-	1.00	-
**anxiety disorder**	**Yes**	1.25	0.56-2.79	1.85	0.80-4.27	1.72	0.67-4.44	1.59	0.67-3.78	1.88	0.69-5.12	0.78	0.35-1.73	1.19	0.52-2.71	0.83	0.38-1.83	1.66	0.44-6.31
**Comorbid substance**	**No (ref.)**	1.00	-	1.00	-	1.00	-	1.00	-	1.00	-	1.00	-	1.00	-	1.00	-	1.00	-
**use disorder**	**Yes**	2.66	0.85-8.32	1.91	0.57-6.47	**8.29	1.69-40.58	2.51	0.70-9.02	0.99	0.22-4.49	2.80	0.84-9.35	1.36	0.39-4.78	1.75	0.56-5.42	3.69	0.56-24.52

*p < 0.05; **p < 0.01. These p-values indicate a significance of the difference of the odds ratios between categories tested by *χ*2-test.

^a^Antidepressant prescribed.

^b^At least 1 visit with a physician a year.

^c^Antidepressant used for at least 2 months + 4 visits with a physician a year.

^d^At least 1 session of psychotherapy a year.

^e^Antidepressant used for at least 2 months + at least 4 visits with a physician a year or at least 8 sessions of psychotherapy a year or a hospitalization for depressive symptoms lasting for at least 4 days.

^f^A participant discontinued the visits despite adequate treatment plan.

^g^The most recent depressive episode.

^f^ All the variables were entered simultaneously into a logistic regression model.

OR = Odds ratio; 95% CI = 95% confidence interval.

Women had higher odds of having any psychotherapy (OR 2.53, CI 1.04–6.16, P = 0.041) and at least 8 sessions of psychotherapy a year (OR 3.37, CI 1.20–9.46, P = 0.021) (Table 5). In the most intensively treated depressive episode the same kind of trend towards womens’ higher odds of having psychotherapy was seen, but the p-values were just above the significance limit (Additional file 4: Table S3). Concerning age, those aged 25–29 had the lowest odds of having any medication (OR 0.20, CI 0.07–0.57, P = 0.003) and guideline-concordant medication (OR 0.30, CI 0.10–0.92, P = 0.035) as well as at least one visit with a physician (OR 0.18, CI 0.05–0.63, P = 0.008) compared to the youngest age group. None of these factors explained the final outcome of minimally adequate treatment (Table 5).

The explanatory factors above were also used in the logistic regression model to examine treatment dropout. In the multivariate analysis persons who had graduated from high school had a lower odds of dropping out of treatment (OR 0.15, CI 0.03–0.86, P = 0.033). Lifetime history of suicide attempts was associated with increased odds of treatment interruption (OR 6.73, CI 1.10–41.17, P = 0.039) (Table 5). The latter difference was not statistically significant in the multivariate analysis concerning the most intensively treated depressive episode (Additional file 4: Table S3).

## Discussion

Compared to previous Finnish studies, treatment of depressive disorders has improved among young adults [12,13,43]. A similar trend was seen in Canada [44]. In our study of young adults with depressive disorders, 76.1% had had some kind of contact because of their depressive symptoms with the health care system during the most recent depressive episode and 40.9% had received minimally adequate treatment, as based on international guidelines. In most studies concerning the treatment adequacy of depressive disorders the proportion has been much lower. In the National Comorbidity Survey Replication study, only 21.7% of all respondents meeting the 12-month major depressive disorder criteria received adequate treatment in the year of the interview [6]. Gonzalez et al. found a very similar figure (21.3%) when he investigated the use of guideline-concordant depression care among adults with 12-month MDE in the United States, though the definition of adequate treatment was less strict than ours in that they only required four psychotherapy visits [45]. In Canada, Duhoux et al. found that 29.1% of subjects with MDE during the 12 months preceding the survey had received minimally adequate treatment according to criteria which were close to ours [14]. However, detailed comparisons between countries are difficult because of lack of relevant and valid comparable data on mental health services [46].

The high proportion of adequate treatment in our study may be explained partly by our versatile information search. We gathered information also from case records, while most surveys have used only interviews, which may be influenced by memory bias. Our participants were young and we investigated the most recent depressive episode, and these factors also support the conclusion that memory bias is not a major problem in this study. A slight increase in our figure may be caused by our definition of a visit with a physician, which also included consultations by telephone and by another health care professional. In addition, we defined at least four days of hospitalizations as adequate. These cases, however, were few (10 participants) and half of them had received adequate treatment also in outpatient care.

None of the factors chosen were related to the overall adequacy of treatment in a multivariate analysis. This suggests that services for the treatment of depression are not functioning efficiently: people with more severe or comorbid symptoms did not receive adequate treatment more often than others. This is alarming, because according to previous studies both severity of depression and comorbidity are associated with worse long-term outcome of depression [47,48]. In terms of health equity, it is encouraging that sociodemographic factors were not associated with receiving minimally adequate treatment. However, in a bivariate analysis, the duration of depressive episode was related to treatment such that participants whose episode had lasted over a year received more often any care and guideline-concordant care in regard to all the various aspects of treatment as well as minimally adequate treatment. This is in line with previous epidemiological studies that have shown that longer duration of depressive episode is related to treatment referral [6,49-51]. In a study among adolescents and young adults, Wittchen et al. found that participants with dysthymia and recurrent depression had sought help more often (46% and 40%) than participants with a single episode of depression (24%) [8]. Wang et al. found that delays in initial help-seeking is a pervasive problem worldwide, with median delays in making contact ranging from 1.0 to 14.0 years for mood disorders [52]. Thus, a likely reason for the lower extent of treatment for those who have suffered for a shorter period is that they have not yet sought help. This may also be one explanation for a higher estimate of treatment adequacy in our study compared to other previously mentioned studies: we investigated the most recent lifetime depressive episode whereas many other studies have looked at depressive episodes within the last 12 months. It is likely that in other studies, all participants with depressive disorder had not yet sought help though they would do so in the future.

It has previously been shown that the severity of depression is also associated with treatment referral [6,7,43,50,53,54]. In this study, having a diagnosis of MDD represented the severity of illness and was associated with having some forms of treatment, especially guideline-concordant physician visits. So it seems that physicians tend to meet the most complicated cases more often than others, as an association between comorbid substance use disorder and physician visits was also found. This suggests that referral to treatment works better for these groups with more serious illness and comorbidity, although they did not receive minimally adequate treatment more often than other participants with depressive disorder.

Women more often received guideline-concordant psychotherapy than men and this difference was statistically significant even after adjusting for other factors. In the multivariate analysis, female gender was also related to having any psychotherapy and having visited a physician. Unlike in our study, Hämäläinen et al. found that female gender was associated with the use of antidepressants but not with psychological treatment among participants with MDD in the Health 2000 adult sample [43]. However, many previous studies have similarly found a relation between female gender and more frequent use of psychotherapy [55,56], a greater likelihood to use any 12-month mental health services [7,57] and guideline-concordant treatment from mental health specialists [54]. It has been suggested that women are more likely to seek, accept and continue treatment because of their reduced perception of stigma and better ability to translate feelings of distress into conscious recognition of having emotional problems [58].

The annual prevalence of antidepressant use and long-term treatment increased between 1994 and 2003 in Finland [59]. We found in our study that 46.9% of subjects with MDD received some antidepressant pharmacotherapy and 36.1% used medication at least for two months. In the adult sample of the Health 2000 study, only 24% of individuals with MDD were currently receiving antidepressants [43]. In the sample of adolescents and young adults from the Finnish Health Care Survey 1996, only 14% of subjects with MDE reported recent use of antidepressants [13]. According to these figures use of pharmacotherapy has increased recently in Finland and is more frequent among young people than previously.

In our study group, 15.7% of participants dropped out from treatment. In previous studies the rates of disengagement from mental health services have varied from 4–46%, depending on the study setting, service type, and definition of engagement used [60,61]. Our definition of dropout was quite strict, requiring a proper treatment plan. Pinto-Meza et al. studied treatment dropout among patients with depression and anxiety in Europe and found a dropout rate of 14% during a 12-month period [19], which is in line with our figure. In the bivariate analysis we found an association between dropouts and lower education, suicide attempts and comorbid substance use disorder, of which lower education and suicidality remained statistically significant also in the multivariate analysis. It is noteworthy that according to the bivariate analysis, groups with less education and substance use disorder had more often visited a physician at least once. Of these factors, substance use disorder remained significant also after adjustment. So it seems that these problematic subgroups with less education, suicidality and substance use disorder—who are otherwise at risk of social exclusion—are offered treatment but do not receive the intended care because they often drop out.

### Strengths and limitations

The two-phase study design enabled us to conduct SCID-I interviews requiring clinical judgment. These were complemented by case records from mental health treatment contacts. Thus the final diagnostic assessment was based on all the available information, which is exceptional in population-based studies and a key strength of the present study. Using multiple sources of information also in regard to treatments compensated for any possible effect of recall bias. On the other hand this means that our results are not directly comparable with the results of most previous surveys using only information collected by interviews. However, the percentage of minimally adequate treatment was only slightly smaller (38.6%) when we excluded those patients who had not been interviewed.

The limitation of a two-phase study design is attrition [62]. In this study, nonresponse occurred in both the questionnaire and the interview. Nonresponse in the MEAF questionnaire depended on age, gender, and education, but not on self-reported mental health disorders at the baseline survey. Persons with a lifetime hospital treatment for mental health problems returned the questionnaire less often, but this was compensated for by medical records that we obtained. These factors were also most strongly related to attrition in the interview. None of the scores in any of the questionnaire screens we used for the mental health interview differed between interview participants and non-participants. More detailed analysis of nonresponse is presented elsewhere [24]. Another significant limitation was the small size of study sample, which led to low statistical power and wide confidence intervals, particularly in the logistic regression analysis. Furthermore, correction for multiple testing was not done.

A few concerns are related to missing values in some of our variables. We rated dropout from treatment only if a treatment strategy was adequate according to the case records. Since the case records were not always comprehensive in this respect, the variable concerning treatment dropouts contains many (27) missing values. We faced the same problem approximating the duration of the depressive episode (21 missing values), which was done retrospectively based on information in the interview and case records.

A psychotherapeutic session was defined as a visit with a psychiatrist, psychologist or psychotherapist in all settings or other professional in a psychiatric clinic. Therefore, according to our definition the professional was not necessarily a psychotherapist and we could not evaluate the duration of sessions. Thus psychotherapy in this article means psychosocial support broadly, and we cannot draw conclusions on the availability of actual psychotherapy on the basis of this study. It is also remarkable that minimally adequate treatment only signifies that the minimum criterion for treatment adequacy is reached; it does not mean optimal treatment, which would mean that every effort according to depression treatment algorithms was made in order to achieve full symptomatic remission [63].

The Mental Health in Early Adulthood in Finland (MEAF) study was done in 2003–2005 i.e. about ten years ago. After the years of the survey there have been changes in the health care system as well as medication in Finland, which means that figures of minimally adequate treatment may be different today. Probably, the situation has improved: based on official statistics, the use of antidepressants and the number of psychiatric outpatient visits have increased.

## Conclusions

A lack of adequate treatment of depressive disorders is an ongoing problem, although our results on treatments among young adults are better than in most previous studies and encouraging in this respect. Delays in help-seeking and discontinuation of treatment seem to create a barrier to proper care. It is alarming that dropout is related to individuals with less education and suicidality, who are otherwise also persons at greatest risk of complications and social exclusion. These groups present a challenge to future health care and more efforts are needed to outreach and motivate them to receive effective treatment.

## Additional files

Additional file 1:
**Methods.**

Additional file 2: Table S1. Sociodemographic factors, treatments received and dropouts during the most intensively treated depressive episode.
Additional file 3: Table S2. Disorder-spesific factors, comorbid psychiatric disorders, treatments received and dropouts during the most intensively treated depressive episode.
Additional file 4: Table S3. Logistic regression models of variables associated with treatments received and dropout during the depressive episode.
