# Supplementary material for: Treatment received and treatment adequacy of depressive disorders among young adults in Finland
Source: BMC Psychiatry. 2015 Mar 11;15:47. doi: 10.1186/s12888-015-0427-8 (PMC4364633; doi:10.1186/s12888-015-0427-8)
Supplement: Additional file 1: — Methods. [file 12888_2015_427_MOESM1_ESM.doc]

**Additional file 1:**

**Construction of the sample:**

**Health 2000 study design**

The target population of the Health 2000 Survey consisted of individuals aged 18 or over and living in mainland Finland in July 1^st^, 2000. In addition to the household population, people living in institutions were included. The Autonomous Territory of Åland Islands was excluded, as were people living on islands not accessible by road. [1]

The basic sampling design was a stratified two-stage cluster sampling design. The aim was to select a sample that properly reflected the main demographic distributions of the Finnish population and covered the whole country, while allowing data collection at reasonable cost. In the first sampling stage 80 health centre districts were sampled. The 15 largest health centre districts were selected with probability 1. The remaining 234 health centre districts were then stratified by the five university hospital regions. This was followed by the selection of 65 health centre districts. In the second sampling stage, the Social Insurance Register was used as the sampling frame for the people resident in the selected 80 health centre districts. The ultimate sampling units were persons who were selected by systematic sampling from the health centre districts. The process is described in detail in the Methodology report of the Health 2000 study which also shows the geographical distribution of the selected health centre districts [1].

Assessment of the young adult sample, carried out in 2001, involved the use of an interview and a questionnaire to gather information on sociodemographic factors, childhood and school experiences, health, use of medication and health services, and lifestyle. Although there were questions related to mental health, a structured diagnostic interview was not conducted. In contrast, the adult sample was interviewed with the Composite International Diagnostic interview [2]. Because of this, the Mental Health in Early Adulthood was done.

**Mental Health in Early Adulthood design**

The Mental Health in Early Adulthood (MEAF) study had a two-phase study design. In the first phase, a questionnaire was sent to all members of the young adult sample, excluding those who had died (n=5) or refused further contacts (n=26). Based on responses to the questionnaire and on information from the Finnish Hospital Discharge Register, we selected a part of the sample to the interview (second phase).

**MEAF mental health screen**

MEAF mental health screen was designed to capture all current and lifetime mental disorders. Therefore, it consisted of several elements and the overall aim was to be as inclusive as possible, but also to allow identification of people with a low likelihood of any mental disorder, from whom only a random sample was invited to the interview.

The individual cut-offs for the screens were chosen based on previous Finnish research or the recommended cut-offs in the original publications. The GHQ-12 cut-off, which was higher than in many other studies, was set based on previous results from the baseline Health 2000 survey [3, 4]. The CAGE cut-off was high (>2) because a previous study found that about 30% of Finnish young adults receive a score of >1 in CAGE which often is used as a cut-off [5]. As for K-10, SCOFF and MDQ, we used the cut-offs recommended by the developers of these scales [6-8]. The other screens we used have not been previously used as such. The psychosis screen consisted of a positive answer to any of the CIDI’s psychotic symptom questions except for three that were reported by up to 30 per cent of the sample. For drug use, all people who had reported having used any drug at least 5 times were considered screen-positive. People with a history of suicidal ideation or suicide attempt were invited in the interview, as were all who reported a previous or current treatment contact for mental health problems or who reported that they would have needed one. Finally all people with a hospital treatment for any mental disorder based on the hospital discharge register data were invited in the interview. Overall, 58% of the survey sample was screen-positive. Based on the MEAF questionnaire screen, 768 participants were screen-positive in at least one of the screening questionnaires/questions, and they were all invited in the interview. Of screen-negative participants, 161 (29.4%) out of 548 were invited in the MEAF interview, and 88 (54.6%) participated. A detailed analysis of attrition is presented in the supplementary material of Suvisaari et al. (2009). [9].

**Definition of minimally adequate treatment and dropout:**

**Minimally adequate treatment** was defined as follow:

**Guide-line concordant pharmacotherapy:** use of antidepressant for at least two months and at least four visits with a physician within 12 months

or

**Guideline concordant psychotherapy:** at least eight sessions within 12 months with a psychiatrist, psychologist or psychotherapist in any setting, or any professional in specialty mental health services.

or

at least four days of hospitalization for depressive symptoms.

Criteria concerning medication and psychotherapy / psychosocial support are based on evidence-based guidelines [10, 11] and previous surveys [12, 13] Hospitalizations have not been used as an adequacy criterion in previous surveys. Based on the information from the case records and the fact that evaluation period in Finland is four days, we considered four days sufficient to assess the diagnosis as well as to plan and start the treatment.

We used all available information to determine minimally adequate treatment and its components. We counted the visits and evaluated the duration of medication based on the information in the case records, if they were available. If case records were missing or incomplete (for example in the case of treatment in private sector) we estimated the visits and medication use based on the interview which included questions about the type, duration and frequency of treatment. For example a participant could have told that he had visited a private psychotherapist once a week for a year or used an antidepressant for about half a year.

**Treatment dropout** was rated if the treatment strategy was assessed to be adequate according to the case records but the patient discontinued the visits by his own decision.

Treatment strategy was assessed to be adequate if according to the case records there had been a plan to start or continue psychotherapeutic visits or medication. Also the reason for discontinuation of visits was extracted from the case records. Typically it was written in a scheduled appointment that “a patient did not show up, nor cancelled a visit” and after some missed appointments there could be an epicrisis telling that the patient had discontinued treatment.

References

1. Heistaro S, Kansanterveyslaitos: *Methodology report: Health 2000 Survey:* Helsinki: National Public Health Institute; 2008.

2. Pirkola SP, Isometsä E, Suvisaari J, Aro H, Joukamaa M, Poikolainen K, Koskinen S, Aromaa A, Lönnqvist JK: **DSM-IV mood-, anxiety- and alcohol use disorders and their comorbidity in the Finnish general population--results from the Health 2000 Study.** Soc Psychiatry Psychiatr Epidemiol 2005, **40**(1):1-10.

3. Aromaa A, Koskinen S, Kansanterveyslaitos: *Health and functional capacity in Finland: baseline results of the Health 2000 health examination survey:* Helsinki: National Public Health Institute; 2004.

4. Aalto AM, Elovainio M, Kivimaki M, Uutela A, Pirkola S: **The Beck Depression Inventory and General Health Questionnaire as measures of depression in the general population: a validation study using the Composite International Diagnostic Interview as the gold standard.** Psychiatry Res 2012, **197**(1-2):163-171.

5. Aalto-Setälä T, Marttunen M, Tuulio-Henriksson A, Poikolainen K, Lönnqvist J: **Depressive symptoms in adolescence as predictors of early adulthood depressive disorders and maladjustment.** Am J Psychiatry 2002, **159**(7):1235-1237.

6. Morgan JF, Reid F, Lacey JH: **The SCOFF questionnaire: assessment of a new screening tool for eating disorders.** BMJ 1999, **319**(7223):1467-1468.

7. Hirschfeld RM, Williams JB, Spitzer RL, Calabrese JR, Flynn L, Keck PE,Jr, Lewis L, McElroy SL, Post RM, Rapport DJ, Russell JM, Sachs GS, Zajecka J: **Development and validation of a screening instrument for bipolar spectrum disorder: the Mood Disorder Questionnaire.** Am J Psychiatry 2000, **157**(11):1873-1875.

8. Kessler RC, Barker PR, Colpe LJ, Epstein JF, Gfroerer JC, Hiripi E, Howes MJ, Normand SL, Manderscheid RW, Walters EE, Zaslavsky AM: **Screening for serious mental illness in the general population.** Arch Gen Psychiatry 2003, **60**(2):184-189.

9. Suvisaari J, Aalto-Setala T, Tuulio-Henriksson A, Harkanen T, Saarni SI, Perala J, Schreck M, Castaneda A, Hintikka J, Kestila L, Lahteenmaki S, Latvala A, Koskinen S, Marttunen M, Aro H, Lonnqvist J: **Mental disorders in young adulthood.** Psychol Med 2009, **39**(2):287-299.

10. American Psychiatric Association: **Practice guideline for the treatment of patients with major depressive disorder (revision).** Am J Psychiatry 2000, **157**(4 Suppl):1-45.

11. National Institute for Clinical Excellence: **Management of depression in Primary and Secondary Care.** NICE 2004 [http://www.nice.org.uk].

12. Fernández A, Haro JM, Martinez-Alonso M, Demyttenaere K, Brugha TS, Autonell J, de Girolamo G, Bernert S, Lépine JP, Alonso J: **Treatment adequacy for anxiety and depressive disorders in six European countries.** Br J Psychiatry 2007, **190**:172-173.

13. Wang PS, Lane M, Olfson M, Pincus HA, Wells KB, Kessler RC: **Twelve-month use of mental health services in the United States: results from the National Comorbidity Survey Replication.** Arch Gen Psychiatry 2005, **62**(6):629-640.
