# Supplementary material for: Treatment received and treatment adequacy of depressive disorders among young adults in Finland
Source: BMC Psychiatry. 2015 Mar 11;15:47. doi: 10.1186/s12888-015-0427-8 (PMC4364633; doi:10.1186/s12888-015-0427-8)
Supplement: Additional file 2: Table S1. — Sociodemographic factors, treatments received and dropouts during the most intensively treated depressive episode. [file 12888_2015_427_MOESM2_ESM.doc]

**Additional file 2: Table S1 Sociodemographic factors, treatments received and dropouts during the most intensively treated depressive episode^i^**

|  |  |  |  |  | | | |  | | | | **Guideline-** | |  | | | | **Minimally** | |  | |
| --- | --- | --- | --- | --- | --- | --- | --- | --- | --- | --- | --- | --- | --- | --- | --- | --- | --- | --- | --- | --- | --- |
|  |  |  |  |  | | | | **Visits with** | | | | **concordant** | | **Sessions of** | | | | **adequate** | | **Treatment** | |
|  |  |  |  | **Pharmacotherapy** | | | | **a physician / a year** | | | | **pharmacotherapy^c^** | | **psychotherapy / a year** | | | | **treatment^e^** | | **dropout^f^** | |
|  |  |  |  | **Any^a^** | | **≥2 months** | | **Any^b^** | | **≥4 times** | |  | | **Any^d^** | | **≥8 times** | |  | |  | |
| **Variable** | **Category** |  | **N** | **%** | **N** | **%** | **N** | **%** | **N** | **%** | **N** | **%** | **N** | **%** | **N** | **%** | **N** | **%** | **N** | **%** | **N** |
| **All** |  |  | 142 | 45.8 | 65 | 33.1 | 45 | 68.1 | 94 | 31.9 | 44 | 21.1 | 30 | 61.4 | 86 | 37.1 | 52 | 45.1 | 64 | 18.5 | 22 |
| **Gender** | **Male** |  | 45 | 46.7 | 21 | 38.1 | 16 | 62.2 | 28 | 37.8 | 17 | 26.7 | 12 | 55.6 | 25 | 26.7 | 12 | 44.4 | 20 | 27.0 | 10 |
|  | **Female** |  | 97 | 45.4 | 44 | 30.9 | 29 | 71.0 | 66 | 29.0 | 27 | 18.6 | 18 | 64.2 | 61 | 42.1 | 40 | 45.4 | 44 | 14.6 | 12 |
|  |  | **p^g^** |  | 0.8845 |  | 0.4068 |  | 0.3014 |  | 0.3014 |  | 0.2707 |  | 0.3258 |  | 0.0775 |  | 0.9187 |  | 0.1070 |  |
| **Agegroup** | **<25 years** |  | 31 | 64.5 | 20 | 46.7 | 14 | 80.7 | 25 | 35.5 | 11 | 32.3 | 10 | 73.3 | 22 | 56.7 | 17 | 61.3 | 19 | 19.2 | 5 |
|  | **25-29 years** |  | 53 | 30.2 | 16 | 22.6 | 12 | 52.9 | 27 | 31.4 | 16 | 18.9 | 10 | 58.5 | 31 | 30.2 | 16 | 35.9 | 19 | 18.2 | 8 |
|  | **≥30 years** |  | 58 | 50.0 | 29 | 35.9 | 19 | 75.0 | 42 | 30.4 | 17 | 17.2 | 10 | 57.9 | 33 | 33.3 | 19 | 44.8 | 26 | 18.4 | 9 |
|  |  | **p^g^** |  | **0.0068** |  | 0.0709 |  | **0.0119** |  | 0.8820 |  | 0.2239 |  | 0.3186 |  | **0.0418** |  | 0.0775 |  | 0.9937 |  |
| **Basic** | **Less than high school** |  | 69 | 53.6 | 37 | 37.5 | 24 | 77.6 | 52 | 40.3 | 27 | 26.1 | 18 | 63.2 | 43 | 38.2 | 26 | 49.3 | 34 | 26.3 | 15 |
| **education** | **High school** |  | 67 | 35.8 | 24 | 27.3 | 18 | 56.9 | 37 | 18.5 | 12 | 13.4 | 9 | 57.6 | 38 | 34.9 | 23 | 37.3 | 25 | 5.4 | 3 |
|  |  | **p^g^** |  | **0.0369** |  | 0.2125 |  | **0.0112** |  | **0.0060** |  | 0.0644 |  | 0.5030 |  | 0.6840 |  | 0.1594 |  | **0.0023** |  |
| **Current** | **Employed** |  | 80 | 38.8 | 31 | 26.3 | 20 | 65.4 | 51 | 28.2 | 22 | 15.0 | 12 | 57.7 | 45 | 30.8 | 24 | 40.0 | 32 | 17.2 | 11 |
| **employment** | **Student** |  | 29 | 55.2 | 16 | 37.9 | 11 | 71.4 | 20 | 32.1 | 9 | 24.1 | 7 | 69.0 | 20 | 48.3 | 14 | 51.7 | 15 | 14.8 | 4 |
|  | **Unemployed** |  | 10 | 30.0 | 3 | 22.2 | 2 | 50.0 | 5 | 20.0 | 2 | 20.0 | 2 | 40.0 | 4 | 20.0 | 2 | 20.0 | 2 | 37.5 | 3 |
|  | **Other** |  | 18 | 61.1 | 11 | 52.9 | 9 | 76.5 | 13 | 35.3 | 6 | 33.3 | 6 | 66.7 | 12 | 50.0 | 9 | 55.6 | 10 | 0.0 | 0 |
|  |  | **p^g^** |  | 0.1531 |  | 0.1439 |  | 0.5061 |  | 0.8474^h^ |  | 0.2611^h^ |  | 0.3699 |  | 0.1422 |  | 0.2115 |  | 0.1030 |  |
| **Married or** | **No** |  | 68 | 41.2 | 28 | 28.8 | 19 | 61.5 | 40 | 27.7 | 18 | 22.1 | 15 | 56.7 | 38 | 40.3 | 27 | 44.1 | 30 | 10.5 | 6 |
| **cohabiting** | **Yes** |  | 69 | 47.8 | 33 | 35.4 | 23 | 72.1 | 49 | 30.9 | 21 | 17.4 | 12 | 63.2 | 43 | 32.4 | 22 | 42.0 | 29 | 21.1 | 12 |
|  |  | **p^g^** |  | 0.4336 |  | 0.4186 |  | 0.1974 |  | 0.6862 |  | 0.4923 |  | 0.4395 |  | 0.3371 |  | 0.8050 |  | 0.1233 |  |

^a^ Antidepressant prescribed.

^b^ At least 1 visit with a physician a year.

^c^ Antidepressant used for at least 2 months + 4 visits with a physician a year.

^d^ At least 1 session of psychotherapy a year.

^e^ Antidepressant used for at least 2 months + at least 4 visits with a physician a year or at least 8 sessions of psychotherapy a year or a hospitalization for depressive symptoms lasting for at least 4 days.

f A participant discontinued the visits despite adequate treatment plan.

^g^ The p-values indicate a significance of the difference between categories in a distribution of treatments and dropout tested by χ2- or Fisher's exact test. P-values < 0.05 in boldface.

^h^ Fisher's exact test was used in the analysis.

^i^ A bivariate analysis.
