# Supplementary material for: Treatment received and treatment adequacy of depressive disorders among young adults in Finland
Source: BMC Psychiatry. 2015 Mar 11;15:47. doi: 10.1186/s12888-015-0427-8 (PMC4364633; doi:10.1186/s12888-015-0427-8)
Supplement: Additional file 3: Table S2. — Disorder-spesific factors, comorbid psychiatric disorders, treatments received and dropouts during the most intensively treated depressive episode. [file 12888_2015_427_MOESM3_ESM.doc]

**Table S2 Disorder-spesific factors, comorbid psychiatric disorders, treatments received and dropouts during the most intensively treated depressive episode^i^**

|  |  |  |  |  | | | |  | | | | **Guideline-** | |  | | | | **Minimally** | |  | |
| --- | --- | --- | --- | --- | --- | --- | --- | --- | --- | --- | --- | --- | --- | --- | --- | --- | --- | --- | --- | --- | --- |
|  |  |  |  |  |  |  |  | **Visits with** | | | | **concordant** | | **Sessions of** | | | | **adequate** | | **Treatment** | |
|  |  |  |  | **Pharmacotherapy** | | | | **physician/a year** | | | | **pharmacotherapy^c^** | | **psychotherapy/a year** | | | | **treatment^e^** | | **dropout^f^** | |
|  |  |  |  | **Any^a^** | | **≥2 months** | | **Any^b^** | | **≥4 times** | |  | | **Any^d^** | | **≥8 times** | |  | |  | |
| **Variable** | **Category** |  | **N** | **%** | **N** | **%** | **N** | **%** | **N** | **%** | **N** | **%** | **N** | **%** | **N** | **%** | **N** | **%** | **N** | **%** | **N** |
| **Major** | **Yes** |  | 111 | 47.8 | 53 | 35.5 | 38 | 70.1 | 75 | 34.6 | 37 | 22.5 | 25 | 62.7 | 69 | 39.1 | 43 | 46.9 | 52 | 17.4 | 16 |
| **depressive disorder** | **No** |  | 31 | 38.7 | 12 | 24.1 | 7 | 61.3 | 19 | 22.6 | 7 | 16.1 | 5 | 56.7 | 17 | 30.0 | 9 | 38.7 | 12 | 22.2 | 6 |
|  |  | **p^g^** |  | 0.3718 |  | 0.2482 |  | 0.3544 |  | 0.2068 |  | 0.4407 |  | 0.5455 |  | 0.3610 |  | 0.4208 |  | 0.5794^h^ |  |
| **Duration of**  **episode >1year** | **Yes** |  | 45 | 71.1 | 32 | 61.4 | 27 | 86.1 | 37 | 53.5 | 23 | 42.2 | 19 | 79.6 | 35 | 68.2 | 30 | 71.1 | 32 | 20.5 | 8 |
|  | **No** |  | 82 | 32.9 | 27 | 20.3 | 16 | 61.3 | 49 | 20.0 | 16 | 11.0 | 9 | 50.6 | 41 | 23.5 | 19 | 30.5 | 25 | 14.5 | 10 |
|  |  | **p^g^** |  | **<.0001** |  | **<.0001** |  | **0.0042** |  | **0.0001** |  | **<.0001** |  | **0.0016** |  | **<.0001** |  | **<.0001** |  | 0.4201 |  |
| **Suicide attempts** | **Yes** |  | 17 | 64.7 | 11 | 37.5 | 6 | 88.2 | 15 | 58.8 | 10 | 35.3 | 6 | 76.5 | 13 | 29.4 | 5 | 47.1 | 8 | 53.9 | 7 |
|  | **No** |  | 125 | 43.2 | 54 | 32.5 | 39 | 65.3 | 79 | 28.1 | 34 | 19.2 | 24 | 59.4 | 73 | 38.2 | 47 | 44.8 | 56 | 14.2 | 15 |
|  |  | **p^g^** |  | 0.0949 |  | 0.6897 |  | 0.0573 |  | **0.0109** |  | 0.2003^h^ |  | 0.1740 |  | 0.4815 |  | 0.8606 |  | **0.0024^h^** |  |
| **Comorbid** | **Yes** |  | 46 | 50.0 | 23 | 42.2 | 19 | 80.4 | 37 | 41.3 | 19 | 28.3 | 13 | 69.6 | 32 | 45.7 | 21 | 52.2 | 24 | 27.9 | 12 |
| **anxiety disorder** | **No** |  | 96 | 43.8 | 42 | 28.6 | 26 | 62.0 | 57 | 27.2 | 25 | 17.7 | 17 | 57.5 | 54 | 33.0 | 31 | 41.7 | 40 | 13.2 | 10 |
|  |  | **p^g^** |  | 0.4842 |  | 0.1114 |  | **0.0281** |  | 0.0931 |  | 0.1494 |  | 0.1665 |  | 0.1449 |  | 0.2389 |  | 0.0684 |  |
| **Comorbid** | **Yes** |  | 27 | 59.3 | 16 | 41.7 | 10 | 88.5 | 23 | 50.0 | 13 | 25.9 | 7 | 69.2 | 18 | 30.8 | 8 | 51.9 | 14 | 42.9 | 9 |
| **substance use disorder** | **No** |  | 115 | 42.6 | 49 | 31.3 | 35 | 63.4 | 71 | 27.7 | 31 | 20.0 | 23 | 59.7 | 68 | 38.6 | 44 | 43.5 | 50 | 13.3 | 13 |
|  |  | **p^g^** |  | 0.1181 |  | 0.3250 |  | **0.0135** |  | **0.0278** |  | 0.4972 |  | 0.3651 |  | 0.4561 |  | 0.4313 |  | **0.0037^h^** |  |
| **Comorbid** | **Yes** |  | 12 | 50.0 | 6 | 33.3 | 4 | 90.9 | 10 | 36.4 | 4 | 16.7 | 2 | 58.3 | 7 | 41.7 | 5 | 41.7 | 5 | 18.2 | 2 |
| **eating disorder** | **No** |  | 130 | 45.4 | 59 | 33.1 | 41 | 66.1 | 84 | 31.5 | 40 | 21.5 | 28 | 61.7 | 79 | 36.7 | 47 | 45.4 | 59 | 18.5 | 20 |
|  |  | **p^g^** |  | 0.7588 |  | 1.0000^h^ |  | 0.1735^h^ |  | 0.7437^h^ |  | 1.0000^h^ |  | 1.0000^h^ |  | 0.7612^h^ |  | 0.8044 |  | 1.0000^h^ |  |

^a^ Antidepressant prescribed.

^b^ At least 1 visit with a physician a year.

^c^ Antidepressant used for at least 2 months + 4 visits with a physician a year.

^d^ At least 1 session of psychotherapy a year.

^e^ Antidepressant used for at least 2 months + at least 4 visits with a physician a year or at least 8 sessions of psychotherapy a year or a hospitalization for depressive symptoms lasting for at least 4 days.

^f^ A participant discontinued the visits despite adequate treatment plan.

^g^ The p-values indicate a significance of the difference between categories in a distribution of treatments and dropout tested by χ2- or Fisher's exact test. P-values < 0.05 in boldface.

^i^ A bivariate analysis.
