# Supplementary material for: Treatment received and treatment adequacy of depressive disorders among young adults in Finland
Source: BMC Psychiatry. 2015 Mar 11;15:47. doi: 10.1186/s12888-015-0427-8 (PMC4364633; doi:10.1186/s12888-015-0427-8)
Supplement: Additional file 4: Table S3. — Logistic regression models of variables associated with treatments received and dropout during the depressive episode. [file 12888_2015_427_MOESM4_ESM.doc]

**Additional file 4: Table S3 Logistic regression models of variables associated with treatments received and dropout during the depressive episode ^g, f^**

|  |  |  | |  | | |  | | |  | |  | |  | |  | |  | **Guideline-** | |  | |  | |  | |  | **Minimally** | |  | |  |
| --- | --- | --- | --- | --- | --- | --- | --- | --- | --- | --- | --- | --- | --- | --- | --- | --- | --- | --- | --- | --- | --- | --- | --- | --- | --- | --- | --- | --- | --- | --- | --- | --- |
|  |  |  |  | | |  | | |  | | | **Visits with** | | | | | | | **concordant** | | **Sessions of** | | | | | | | **adequate** | | **Treatment** | | |
|  |  | **Pharmacotherapy** | | | | | | | | | | **a physician / a year** | | | | | | | **pharmacotherapy^c^** | | **psychotherapy / a year** | | | | | | | **treatment^e^** | | **dropout^f^** | | |
|  |  | **Any^a^** | | | | | | **≥2 months** | | | | **Any^b^** | | | **≥4 times** | | | |  | | **Any^d^** | | | **≥8 times** | | | |  | |  | | |
| **Variable** | **Category** | **OR** | | | **95% CI** | | | **OR** | | | **95% CI** | **OR** | **95% CI** | | **OR** | | **95% CI** | | **OR** | **95% CI** | **OR** | **95% CI** | | **OR** | | **95% CI** | | **OR** | **95% CI** | **OR** | **95% CI** | |
| **Gender** | **Male (ref.)** | 1.00 | | | - | | | 1.00 | | | - | 1.00 | - | | 1.00 | | - | | 1.00 | - | 1.00 | - | | 1.00 | | - | | 1.00 | - | 1.00 | - | |
|  | **Female** | 1.51 | | | 0.61-3.72 | | | 0.83 | | | 0.33-2.08 | **4.27 | 1.48-12.36 | | 1.44 | | 0.53-3.93 | | 0.77 | 0.27-2.16 | 2.32 | 0.96-5.60 | | 2.54 | | 0.97-6.63 | | 1.66 | 0.69-3.97 | 0.45 | 0.08-2.41 | |
| **Agegroup** | **<25 years (ref.)** | 1.00 | | | - | | | 1.00 | | | - | 1.00 | - | | 1.00 | | - | | 1.00 | - | 1.00 | - | | 1.00 | | - | | 1.00 | - | 1.00 | - | |
|  | **25-29 years** | **0.19 | | | 0.07-0.56 | | | *0.33 | | | 0.11-0.98 | **0.14 | 0.04-0.51 | | 1.07 | | 0.35-3.25 | | 0.55 | 0.17-1.78 | 0.61 | 0.21-1.78 | | 0.41 | | 0.15-1.18 | | 0.37 | 0.13-1.03 | 0.80 | 0.15-4.35 | |
|  | **≥30 years** | 0.41 | | | 0.12-1.33 | | | 0.69 | | | 0.21-2.29 | 0.28 | 0.06-1.25 | | 0.80 | | 0.23-2.83 | | 0.46 | 0.12-1.82 | 0.62 | 0.18-2.10 | | 0.46 | | 0.14-1.53 | | 0.68 | 0.21-2.14 | 1.05 | 0.14-7.81 | |
| **Age at the onset of** | **Continuous** | 1.03 | | | 0.94-1.13 | | | 1.01 | | | 0.92-1.11 | 1.10 | 0.98-1.23 | | 0.99 | | 0.89-1.09 | | 1.01 | 0.90-1.13 | 0.97 | 0.88-1.06 | | 1.00 | | 0.90-1.10 | | 0.96 | 0.88-1.05 | 1.00 | 0.85-1.17 | |
| **depression** |  |  | | |  | | |  | | |  |  |  | |  | |  | |  |  |  |  | |  | |  | |  |  |  |  | |
| **Basic education** | **Less than high school (ref.)** | 1.00 | | | - | | | 1.00 | | | - | 1.00 | - | | 1.00 | | - | | 1.00 | - | 1.00 | - | | 1.00 | | - | | 1.00 | - | 1.00 | - | |
|  | **High school** | 0.64 | | | 0.29-1.40 | | | 0.76 | | | 0.34-1.73 | 0.49 | 0.20-1.21 | | *0.40 | | 0.17-0.96 | | 0.50 | 0.19-1.30 | 0.88 | 0.41-1.90 | | 0.76 | | 0.35-1.68 | | 0.64 | 0.30-1.37 | *4.46 | 1.06-18.77 | |
| **Major** | **No (ref.)** | 1.00 | | | - | | | 1.00 | | | - | 1.00 | - | | 1.00 | | - | | 1.00 | - | 1.00 | - | | 1.00 | | - | | 1.00 | - | 1.00 | - | |
| **depressive disorder** | **Yes** | 1.39 | | | 0.55-3.53 | | | 1.28 | | | 0.46-3.55 | 1.48 | 0.54-4.03 | | 2.53 | | 0.84-7.65 | | 1.37 | 0.43-4.35 | 1.49 | 0.62-3.58 | | 1.20 | | 0.48-3.04 | | 1.24 | 0.51-3.00 | 0.93 | 0.21-4.03 | |
| **Suicide attempts** | **No (ref.)** | 1.00 | | | - | | | 1.00 | | | - | 1.00 | - | | 1.00 | | - | | 1.00 | - | 1.00 | - | | 1.00 | | - | | 1.00 | - | 1.00 | - | |
|  | **Yes** | 1.86 | | | 0.53-6.57 | | | 0.82 | | | 0.23-2.97 | 1.39 | 0.25-7.82 | | 2.43 | | 0.71-8.33 | | 2.05 | 0.55-7.65 | 1.55 | 0.42-5.72 | | 0.53 | | 0.14-1.95 | | 0.57 | 0.17-1.91 | 0.21 | 0.04-1.08 | |
| **Comorbid** | **No (ref.)** | 1.00 | | | - | | | 1.00 | | | - | 1.00 | - | | 1.00 | | - | | 1.00 | - | 1.00 | - | | 1.00 | | - | | 1.00 | - | 1.00 | - | |
| **anxiety disorder** | **Yes** | 1.14 | | | 0.51-2.57 | | | 1.85 | | | 0.81-4.19 | 2.22 | 0.80-6.20 | | 1.36 | | 0.58-3.18 | | 1.74 | 0.69-4.37 | 1.30 | 0.58-2.94 | | 1.79 | | 0.80-4.01 | | 1.34 | 0.62-2.93 | 0.35 | 0.10-1.15 | |
| **Comorbid substance** | **No (ref.)** | 1.00 | | | - | | | 1.00 | | | - | 1.00 | - | | 1.00 | | - | | 1.00 | - | 1.00 | - | | 1.00 | | - | | 1.00 | - | 1.00 | - | |
| **use disorder** | **Yes** | 2.34 | | | 0.74-7.41 | | | 1.36 | | | 0.42-4.42 | **12.65 | 2.06-77.45 | | 2.04 | | 0.62-6.73 | | 0.80 | 0.22-2.96 | 2.28 | 0.69-7.52 | | 1.10 | | 0.33-3.69 | | 1.64 | 0.55-4.94 | 0.24 | 0.04-1.36 | |

*p<0.05; **p<0.01; ***p<0.001. These p-values indicate a significance of the difference of the odds ratios between categories tested by χ2-test.

^a^ Antidepressant prescribed.

^b^ At least 1 visit with a physician a year.

^c^ Antidepressant used for at least 2 months + 4 visits with a physician a year.

^d^ At least 1 session of psychotherapy a year.

^e^ Antidepressant used for at least 2 months + at least 4 visits with a physician a year or at least 8 sessions of psychotherapy a year or a hospitalization for depressive symptoms lasting for at least 4 days.

^f^ A participant discontinued the visits despite adequate treatment plan.

^g^ The most intensively treated depressive episode

^f^ All the variables were entered simultaneously into a logistic regression model.

OR = Odds ratio; 95% CI = 95% confidence interval.
